# Re-use of research data in the social sciences. Use and users of digital data archive

**DOI:** 10.1371/journal.pone.0303190

**Published:** 2024-05-10

**Authors:** Elina Late, Michael Ochsner

**Affiliations:** 1 Faculty of Information Technology and Communication Sciences, Tampere University, Tampere, Finland; 2 Swiss Centre of Expertise in the Social Sciences, University of Lausanne, Lausanne, Switzerland; University of Tennessee at Chattanooga, UNITED STATES

## Abstract

The aim of this paper is to investigate the re-use of research data deposited in digital data archive in the social sciences. The study examines the quantity, type, and purpose of data downloads by analyzing enriched user log data collected from Swiss data archive. The findings show that quantitative datasets are downloaded increasingly from the digital archive and that downloads focus heavily on a small share of the datasets. The most frequently downloaded datasets are survey datasets collected by research organizations offering possibilities for longitudinal studies. Users typically download only one dataset, but a group of heavy downloaders form a remarkable share of all downloads. The main user group downloading data from the archive are students who use the data in their studies. Furthermore, datasets downloaded for research purposes often, but not always, serve to be used in scholarly publications. Enriched log data from data archives offer an interesting macro level perspective on the use and users of the services and help understanding the increasing role of repositories in the social sciences. The study provides insights into the potential of collecting and using log data for studying and evaluating data archive use.

## Introduction

In the context of the Open Science agenda and the Responsible Research and Innovation movement, nations and organizations have put a lot of effort in building research infrastructures for supporting scholars in open science practice. Research data archives (also referred as data repositories) are part of the infrastructure and their aim is to capture and share digital research datasets. Archiving digital research data aims for improving the quality of research, and for economical savings assuming that data once archived will be useful and used by others [1]. Interest in facilitating data sharing and re-use is high, which is evident in funding agencies’, research organizations’, publishers’ and archives’ efforts in drafting policies regulating data sharing and management [2]. Also, data openness and sharing are increasingly important factors in the evaluation of impact, concerning both research infrastructures and scholars [3, 4].

In the social sciences there is a long tradition in re-using time-series datasets such as those by the World Bank or OECD. However, in the era of open science, data sharing has widened its use to individual scholars uploading their data, which most likely form most of the contents in the digital data archives. Yet, despite the massive financial and intellectual investments, it is still unclear how extensively, by whom and for what purposes research datasets are downloaded from the archives [5]. The proposed benefits of open data will be materialised only fully if the available data are used or re-used by others [5]. Also, the importance of creating quantitative metrics for evaluating the impact of research infrastructures is widely recognized [6].

Will it be possible to realise the optimistic promises of open responsible science when the social sciences go digital? While open research data and data infrastructures have drawn a lot of attention, is there a demand for open data, do differences in re-use exist across types of data, how broad is the base of potential users and where is potential to develop and what service portfolios to be developed? Answering to these questions is vital to understand the evolving knowledge creating practices, the impact of open data and the development of open science and its implementation in research practice. Additionally, this information is important for the archives to better understand the potential needs of their user base. Most of the earlier work has based on self-reported data re-use and focused especially on the experiences and needs of scholars [e.g. 7–12]. However, before data citation practices are fully formalized in social sciences, log data and number of downloads are useful to measure the frequency of data re-use [5, 13, 14]. Also, Khan, Thelwall and Kousha [12] call for more comprehensive disciplinary information about repository uptake for enhancing sustainable data sharing.

By now, only very few studies relying on user log data gathered from the social science archives exist. For example, Borgman and colleagues analysed user log data to identify data re-use in the Dutch interdisciplinary data archive DANS [5] using number of downloads and users. Focusing on data re-use in the social sciences, Late and Kekäläinen [15] studied the use of the Finnish research data archive in more detail based on enriched log data. Applying their methodology using enriched log data we study the use of Swiss data repository, FORSbase, that archives both qualitative and quantitative social science research data. Our study supplements the findings by Late and Kekäläinen [15] by providing comparative evidence from another context. We investigate whether there is a demand for open data in the social sciences and address the following research questions:

1. How many times and by how many users are datasets downloaded from the FORSbase?
2. What type of datasets are downloaded from the archive most often?
3. What roles do the users of the archive represent?
4. For what purposes are datasets downloaded?

The article is structured as follows. First, we present related literature concerning open data, data archives and data re-use in the social sciences. We will then describe the research setting and present the results, which will be discussed before being put into the policy and research practice context to draw conclusions.

## Background

### Research data and data archives in the social sciences

The European Commission [16] defines research infrastructures as “facilities that provide resources and services for research communities to conduct research and foster innovation” (p. 1). Research data archives are thus part of the infrastructure supporting and enabling open science by storing, managing, and disseminating research data by public (or private) funding without a fee for the users. Although, studies have shown that many scholars rely on their personal data storage for sharing data [12, 17], there is a long-standing tradition of using and providing open research data and having large data repositories in the social sciences [18]. International organisations like the World Bank, International Monetary Fund, Freedom House, the OECD or EUROSTAT have provided valuable data for social scientists for decades just as well as national public statistical offices [see e.g., 19–21]. Furthermore, for more specific data, national and international data infrastructures, such as the General Social Survey in the US since 1972, the World Values Survey, the European Values Study, the International Social Survey Programme, or Inter-university Consortium of Political and Social (ICPSR), have been offering rich datasets in open access to social scientists [see, e.g., 22, 23]. Also, individual scholars or teams generated and shared data, such as the Democracy Index [24], the Polity Project [25], or the World Inequality Database [26]. Social science data archives providing a hub for sources for secondary analyses have been established in the 1960ies in the US as well as in Europe [18, 27] and for example, the CESSDA, Consortium of European social science data archives, exists since 1976 [27]. Established data archives, provide support and curation for long-term data preservation for the entire data life cycle and tools for data search [28, 29].

However, while especially international comparative quantitative social science has this long-standing tradition, in other sub-fields like psychology it has been usual that data and measurement instruments were part of a business model and available in closed access. Qualitative social science does not look back on a similar tradition of sharing data even though in 1994, the Qualitative Data Archival Resource Center has been established at the University of Exeter to foster re-use of qualitative data [30]. However, this policy-based request has resulted in a heated debate whether it is ethical to share qualitative data because data are potentially sensitive [30, 31]. The shift to open science in the STEM fields has changed the attention of policy makers and put pressures on those sub-fields in the social sciences where open data sharing has not yet been part of the tradition and, at the same time, opened new opportunities and increased reputation of the shared existing data infrastructures.

Research data has thus been seen as theory-laden concept with a long history [5]. Data can take different forms in different disciplines and a particular combination of interests, abilities and accessibility determine what is identified as data in each instance [32]. Borgman [33] defines data as “entities used as evidence of phenomena for the purposes of research or scholarship” (p. 25). Data are not seen only as by-products of research but as research outputs, valuable commodities, and public objects [1]. Data in the social sciences can remain relevant for analysis for a long time as societal developments and historical perspectives can offer new opportunities of, and approaches to, analysis of historical data to researchers.

### Re-use of research data in social sciences

Open access to research data is an essential aspect in open science because, among others, it facilitates the verification of given results and enhances the effectiveness of research by the re-use of data. However, also negative aspects of data re-use have been identified, such as narrowing the scope and increasing the bias of research [34, 35] and leading to injustice in work division, i.e., when data collectors document and share their data, others may take just advantage of the work accomplished by others, as data stewardship is not acknowledged yet [36]. Furthermore, not all kinds of data can be opened due to data protection and ethical principles [37]. This is a frequent issue in the social sciences and earlier studies have claimed relatively low levels of data sharing and re-use [38–41]. However, some data are frequently re-used in the social sciences as for example the open data published by the European Social Survey led to at least 5000 scientific English language publications between 2003 and 2020 [42].

The whole concept of data re-use needs to be understood far more deeply. Re-use of data can mean for example re-using data to reproduce research, to re-use data independently or to integrate data with other data [12, 33]. Re-using a dataset in its original form can be difficult, even if adequate documentation and tools are available, since much must be understood about why the data were collected and why various decisions about data collection, cleaning, and analysis were made [33, 43, 44]. Combining datasets is far more challenging, as extensive information must be known about each dataset if they are to be interpreted and trusted sufficiently to draw conclusions [45].

By now several studies have analysed scholars needs, experiences and perceptions of data re-use relying on surveys and interviews [7–12, 46, 47]. In a recent survey [12] almost half of the respondents representing social sciences reported re-using data. However, there was some variation between research fields. Data re-use was more frequent by experienced scholars and by those sharing their data. When selecting data for re-use, scholars consider proper documentation, openness, information on usability of data, availability of data in a universal standard format and evidence that the dataset has an associated publication as important factors [12].

Social scientists re-using data value data that are comprehensive, easy to obtain, easy to manipulate, and credible [46]. Identified obstacles for data re-use are, for example, barriers to access, lacking interoperability and lack of support [47]. Faniel, Frank and Yakel [9] identified ICPSR’s data users’ information needs in 12 contexts relating to how data was originally produced, about the repository it has been archived and about the previous re-use of data. They argue that scholars representing different disciplines have distinct differences in the types of information desires, that should be considered in service development. For example, information about missing data was important for the social scientists. Studies focusing on data re-use by novice scholars emphasize the importance of details about the data collection and coding procedures and peer support for data use [8]. Re-using data may contribute to the knowledge creating skills of junior scholars and foster them to socialize to their disciplinary communities [48].

Studies have also focused on how data is searched [49, 50] and witnessed scholars struggling with finding datasets to re-use [12, 51]. Most typically, data is found from relevant papers, conducting web searches, and searching from disciplinary and interdisciplinary data archives [12]. Recently, Lafia, Million and Hemphill [52] studied data search basing their analysis on usage data from ICPSR website. They identified three user paths for navigating the website: direct, orienting, and scenic. Direct and scenic paths targeted dataset retrieval, as orienting paths aimed gathering contextual information. They argue that data archives should support both direct and serendipitous data discovery.

Only a few studies have investigated the use of data archives in the social sciences relying on log data. Borgman and colleagues studied the use of the Danish Data Archiving and Networked Services (DANS) using transaction logs, documentation, and interviews, and showed that communities of data infrastructures can be amorphous and evolve considerably over time [5]. They argue that trust plays an important role in the re-use of a dataset collected by someone else and the reputation of the hosting archive and organizations responsible for the curation process are important elements in trust creation.

Late and Kekäläinen [15] studied the use and users of the Finnish research data archive for social sciences by analysing user log data between 2015 and 2018. According to their study, most datasets were downloaded at least once during the time frame and a clear majority of the downloaded data were quantitative. They discovered that the datasets from the archive were downloaded most often for the purposes of studying or master’s or bachelor’s theses. One fifth of the downloading’s were made for research purposes. Similarly, Bishop’s and Kuula-Luumi’s study [53] about the re-use of qualitative datasets showed that data was downloaded for studying, master’s theses, teaching and research, indicating that data re-use is even less prevalent for qualitative studies. According to Late and Kekäläinen [15] the most typical downloaded dataset was survey data. The Finnish research data archive was most often used by social scientists from Finnish universities. However, there were users from other European countries and even from outside Europe and other organizations. Borgman and colleagues [5] argue that user behaviour tends to correlate with existing data practices in a field, and archives tend to be tailored accordingly. However, the results by Late and Kekäläinen [15] showed that users of the archive for social sciences data represented all major disciplines. Thus, data practices in several fields must be considered when developing the services.

## Research setting

### The context: FORSbase

The research data archive investigated in this study is FORSbase. FORS is the Swiss Centre of Expertise in the Social Sciences that offers data and consulting services in social sciences, conducts national and international surveys, and offers data and research information services to researchers and academic institutions [54]. FORSbase was the archive for research projects and research data in the social sciences in Switzerland managed by FORS. It was established in 1992 and was replaced in December 2021 by SWISSUbase (https://www.swissubase.ch/) based at the same institution and issued in collaboration with several partners that includes the functions of FORSbase but serves as the national data repository across disciplines in Switzerland.

Research data from FORSbase and SWISSUbase can be accessed from the online catalogue (https://forsbase.unil.ch/ and https://www.swissubase.ch/). The catalogue is available in English, German and French. Datasets are downloadable free of charge, but users are required to register before downloading datasets. The database has a special structure: It is centred around research projects. Each project can have several datasets and each dataset can have different versions, while only the latest available version is downloadable.

The FORSbase and SWISSUbase data services follow FAIR data principles [1] and have obtained the official certification of CoreTrustSeal. The CoreTrustSeal is a community based non-profit organization promoting sustainable and trustworthy data infrastructures. FORSbase is a member of CESSDA. The change from FORSbase to SWISSUbase does not have any impact on our analysis and its conclusions because the FORSbase service is integrated in SWISSUbase. The main difference is that the services have been upscaled to accommodate research data and projects from other disciplines (and transdisciplinary research).

### Data collection and analyses

The study is based on quantitative user log data that was collected from FORSbase for the time window from 29.2.2016 to 9.2.2020. This time window represents the full user data available for FORSbase since its rebuild in 2016 until the time of the start of our project. The log data contains information about the number of downloads and downloaded datasets. The data is enriched with a) project information data collected from the database and b) data coming from the registration survey that users have to fill in when downloading data. The project information entails information about the archived datasets such as the dataset type. Registration survey data entails information about the users including their role and purpose of data use. Each time a person downloads data from FORSbase, this information is collected.

**Fig 1:**
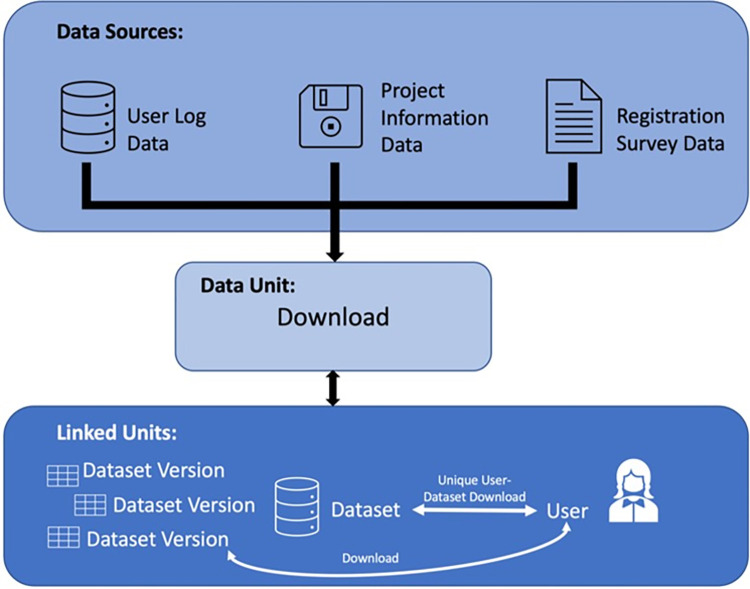
Data structure.

The data is structured as follows (see Fig 1): the main unit is a download; downloads are cross-nested across datasets and users (a user downloading a dataset creates thus a unique download). Each download also points to the version of the dataset that has been downloaded. The raw number of observations (downloads) in the data was 6661. Removing downloads from the dataset made for testing purposes by the FORSbase team resulted in 6656 observations.

The process continued with variable selection and coding. Nine variables analysed in this study are presented in Table 1 along with the research question(s) the variable is used to address. Information for variables 1 to 4 is collected automatically whereas information for variable 5 is constructed in two steps, the name being drawn automatically from the database and then assigned a type of dataset manually from the project information data in the FORSbase online catalogue. Information for variables 6 to 9 is asked from the users in a survey format during registration and when downloading the data.

**Table 1:** Analysed variables and their relationship with research questions.

	Variable	Analyses	Research question
1.	Date of the download	Distribution of downloads per year Average number of downloads per day, month, and year	RQ1
2.	ID number of the user	Number of active users (downloaders) Average number of downloads per unique users	RQ1
3.	ID number of the dataset	Number of unique downloaded datasets Version of the dataset	RQ1, RQ2
4.	Type of dataset	Share of qualitative or quantitative datasets	RQ2
5.	Name of dataset	Name of 10 most downloaded datasets	RQ2
6.	The role of the user	Distribution of users by role	RQ3
7.	Use purpose	Distribution of downloads for teaching and research	RQ4
8.	Research description (open ended question)	Categorisation of the use purposes other than teaching/research, type of studying purpose	RQ4
9.	Is publication expected (yes/no)	Distribution of downloads aiming and not aiming for publication	RQ4

To identify how many times and by how many users are datasets downloaded from FORSbase (RQ1), we analyse the download date, user id, and dataset id (Table 1, variables 1–3). Concerning the number of downloads, we analyse *the full number and share of downloaded datasets* and *unique user-dataset downloads* (Table 1, variable 3) to control for downloading dataset updates and to exclude duplicate downloads. By analysing the unique dataset downloads we can identify whether the same user downloaded the same dataset twice or two versions of it. Concerning the number of users, we analyse the average number of downloads for the *registered users* and the *active users* (Table 1, variable 2). Registered users are those who have registered to FORSbase for archiving and downloading data. The number of registered users was asked from the archive personnel in time of the data collection in 2020. Active users are those who downloaded data during the time window of the data collection. Each user is identified in the data with a unique user ID number automatically provided by the system during registration (Table 1, variable 2).

To identify what type of datasets are downloaded from the archive most often (RQ2) we use *the id of the dataset*, *the type of dataset (quantitative or qualitative data) and the name of the dataset* (Table 1, variables 3, 4, 5). The name of the downloaded dataset (Table 1, variable 5) was also used to study the 10 most downloaded datasets in more detail. For these datasets, information (i.e., descriptive details) were traced from the FORSbase online catalogue.

To analyse what roles do the users of the archive represent (RQ3), we use *the role of the downloading user* (Table 1). Originally, users were provided a list of 11 roles from which they selected the most suitable one. For the analyses, some categories were combined to form a shorter list of seven different roles (i.e., student, doctoral student, lecturer/post doc, professor, other research/project manager, teacher, and non-academic).

Finally, to identify for what purposes datasets are downloaded (RQ4), we use information on *the use purpose of the data*, *the research description* and whether *a publication is expected* (Table 1, variables 7,8, 9). When users were downloading datasets from FORSbase, they were asked whether the dataset was downloaded either for research or for teaching purposes (Table 1, variable 7). Although these categories did not serve well for the students downloading datasets for their course work, they were forced to choose between the two options. Therefore, for the means of this study, a new use purpose type “studying” was constructed manually in two steps. First, all the users that identified themselves as students were identified from the data (Table 1, variable 6). In the second step, the coding was assigned by thoroughly reading the research descriptions (Table 1, variable 8) written by the students to find out the purpose of the download. Based on these descriptions we also categorised the sub-type of studying purpose if possible (e.g., bachelor theses, master’s theses). However, the research description was asked only for those downloads where the users were indicating *research* (Table 1, variable 7) as the purpose for the download. Consequently, this information is missing for the downloads where users indicated *teaching* as purpose. Obviously, this applies also to students who had selected *teaching* as use purpose. These were categorised as *studying* as we assume that students do not teach yet but chose *teaching* as there was no option for studying. Downloading data for doctoral dissertation were categorized as “research” purpose.

Variable nine (Table 1) was used to study the purpose of *research* use of the dataset by asking whether the user was expecting a publication resulting from the downloaded dataset. This information was asked only for those downloading data for *research* purposes. Thus, this information is missing for the downloads the users indicated *teaching* as the purpose.

For the analyses step the data were gathered into one dataset and analysed with Stata 16. Given that we analyse full data, we do not apply inferential statistics. Whenever we are interested in differences between groups, we apply bootstrapped 95 per cent stability intervals to indicate the precision of the estimates. Differences were then tested also using bootstrapping procedures either with regression models (numbers of downloads per user group) or tests on the equality of proportions [55] for the intention to publish across user groups.

## Results

### Number of dataset downloads

**Fig 2:**
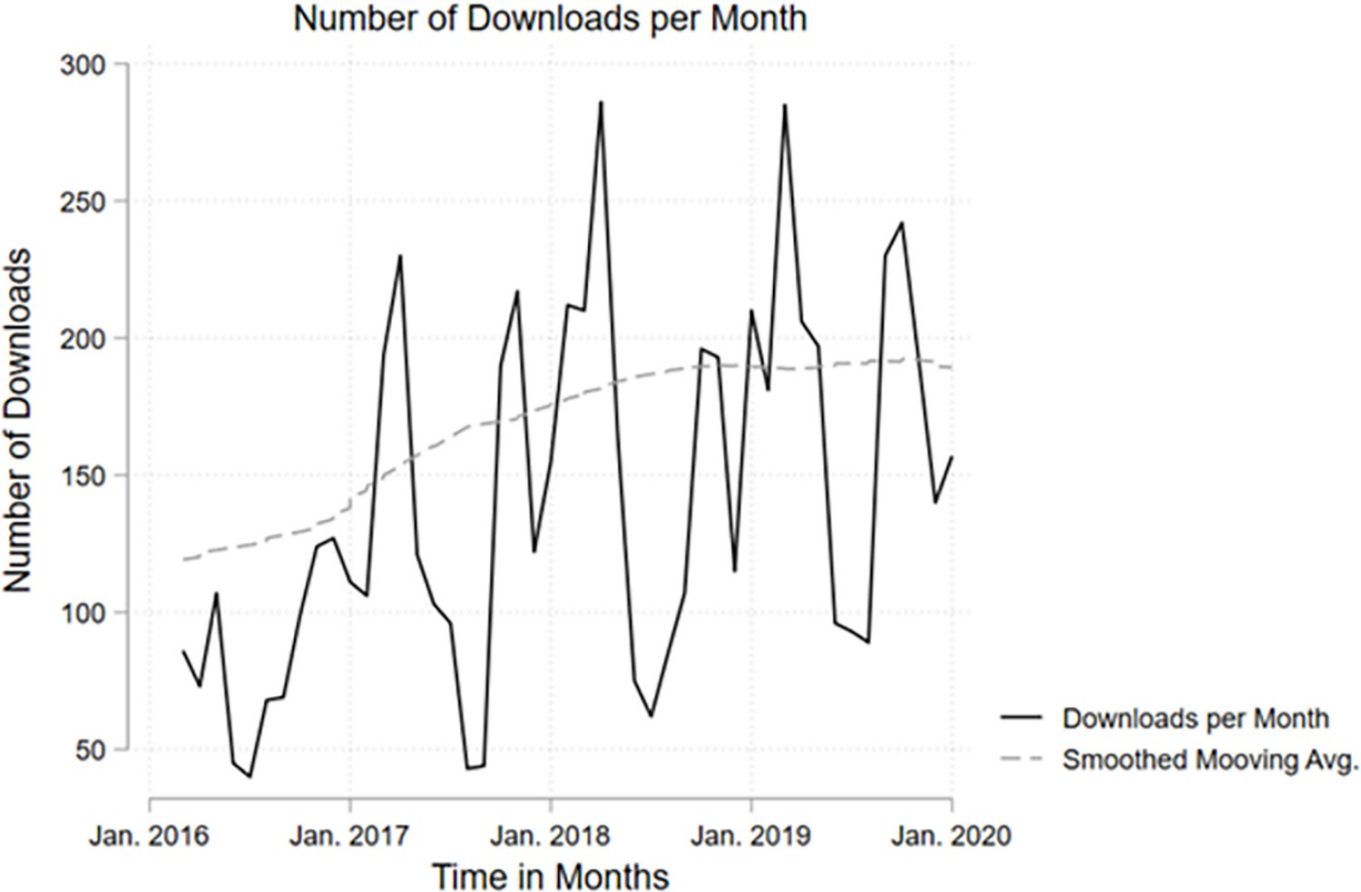
Downloads per month for the time window with full months covered, i.e., March 2016 to January 2020. Smoothed moving average is calculated using weights as suggested in [56].

In February 2020, at the time of our data collection, FORSbase had 6628 registered users. The archive contained 725 datasets, the majority of which were quantitative. Within the time window that covers 49 months, a total of 6656 downloads were made from FORSbase (Table 2). This results in an average 136 downloads per month or 5 downloads per day. When excluding incomplete months from 2016 and 2020 in our dataset, we cover a total of 6593 downloads over 47 months, leading to a mean of 140 downloads per month (*range* = 40–286, *median* = 122). From 2017 to 2018, the number of downloads increased by 18 per cent and from 2018 to 2019 by 16 per cent. The downloads per months show a high volatility as can be seen from Fig 2 that shows the downloads per month for fully covered months, i.e., March 2016 to January 2020, and a smoothed moving average. The figure makes visible an increase of downloads over time with a tendency to stabilise. Note that March, April and October, November show the highest downloads while July and August show the lowest downloads, reflecting semester beginnings for highs and semester break for lows.

**Table 2:** Number of downloads per year.

Year	Frequency	Percent	Avg. per month**
2016*	839	12.6	84
2017	1577	23.7	131
2018	1860	27.9	155
2019	2161	32.5	180
2020*	219	3.3	n/a
Total	6656	100.0	

*Notes*. * The time window does not cover the full year for 2016 (February 29^th^–December 31^st^) and 2020 (January 1^st^-February 9^th^).

** Only full months are taken into account. January-February 2016 and January and February are excluded from the calculations.

Of the 725 datasets archived in FORSbase, 470 datasets were downloaded at least once representing 65 per cent of all archived datasets. One fifth of the downloaded datasets were downloaded once and 13 per cent twice. Consequently, 67 per cent were downloaded three times or more (see Table 3). Datasets, however, can be updated and new versions are released. Users are informed so that they can download the new version. This leads to the fact that some datasets are downloaded more often than others. Additionally, users can download the same dataset twice (e.g., on two different workstations). To control for updates and to have a measure that reflects better the number of times a dataset is used (as opposed to downloaded), we identified duplicates, i.e., if the same user downloaded the same dataset twice or two versions of it. This was counted as one *unique user-dataset download* (see Table 3, columns on the right). Both measures are somewhat imperfect because, on the one hand, regarding the full count measure, a dataset that is published quickly and corrected afterwards will score more downloads than one that is not updated. On the other hand, regarding the corrected measure, it might be that a same user downloads the same data multiple times for different persons, e.g., as teacher and student (a situation that is not compliant to the user agreement) or for different uses. Additionally, it is not clearly defined by the database what a “version” is. It is usually an update of the same dataset, but it could also be used to have a dataset updated with new waves while another dataset would create a new dataset for each new wave added. We did our best to control for the later and try to treat a study (and each wave) as a dataset if archived separately.

**Table 3:** Number of downloads per dataset and unique user-dataset downloads.

Number of Downloads	Frequency	Percentage of Total Archived Datasets	Percentage of Datasets at Least Downloaded Once	Frequency of Unique User-Dataset Downloads	Percentage of Unique User-Datasets Downloads	Percentage of Unique User-Datasets at Least Downloaded Once
0	255	35.2	n/a	255	35.2	n/a
1	101	13.9	21.5	106	14.6	22.6
2	55	7.6	11.7	68	9.4	14.5
3+	314	43.3	66.8	296	40.8	63.0
Total	725	100.0	100.0	725	100	100*

* Column sums exceeding 100% are due to rounding

Table 4 shows that the main download statistics between the two measures differ only slightly. The mean amounts to 9 downloads per data (8 if only unique user-dataset downloads are counted), but the distribution is highly skewed with a first quartile of 0 downloads, a median of 2 downloads and a third quartile of 6 downloads irrespective of how to count dataset-downloads.

**Table 4:** General download statistics.

	Mean	1^st^ Quartile	Median	3^rd^ Quartile	Min	Max
All Downloads	9.2	0	2	6	0	638
Unique User-Dataset Downloads	8.1	0	2	6	0	527

### Type of most downloaded datasets

FORSbase allows the archiving of both quantitative and qualitative data. Qualitative data can be archived since 2017 only. From the 725 datasets, only 15 datasets were archived as qualitative datasets, which corresponds to 2 per cent. Of the 470 datasets that were downloaded at least once, 5 were qualitative datasets (1%). On the level of downloads, the vast majority (98%) of the downloads concerned quantitative datasets. Qualitative datasets were downloaded only 15 times (13 times if we consider only unique user-dataset downloads). Two of which were downloaded once, two twice and one nine times (7 times if only unique user-dataset downloads are counted).

Ten datasets were downloaded more than 100 times (see Table 5). Downloads for these 10 datasets represent almost 40 per cent of all downloads from FORSbase in the given time window. FORS was the collector of eight out of the ten most downloaded datasets. The other two datasets were collected by Swiss universities. The most downloaded datasets were all quantitative and either cumulative datasets or single year issues of longitudinal (cross-sectional or panel) surveys collected at regular intervals. Those surveys can be considered social sciences data infrastructures of national or even international importance and are designed for secondary data analysis.

**Table 5:** Ten most downloaded datasets from FORSbase.

Title of the dataset	Number of downloads	Percentage of total downloads (N = 6656)**	Number of unique user-dataset downloads	Percentage of unique user-dataset downloads (N = 5842)**	Collector*
1. SHP Data Waves 1–19	638	9.6 (8.9–10.3)	527	9.0 (8.3–9.8)	FORS
2. Selects 2015 Post-electoral study	400	6.0 (5.5–6.6)	308	5.3 (4.7–5.9)	FORS
3.CCS Wave II—Cumulative Dataset 2013–2018	268	4.0 (3.6–4.5)	206	3.5 (3.1–4.0)	FORS
4.CCS Wave I—Cumulative Dataset 2005–2013	265	4.0 (3.5–4.5)	212	3.6 (3.2–4.1)	FORS
5. Selects, cumulated file 1971–2015	222	3.3 (2.9–3.8)	185	3.2 (2.7–3.6)	FORS
6. Selects 2015 Panel / Rolling cross-section study	216	3.2 (2.8–3.7)	169	2.9 (2.5–3.4)	FORS
7. TREE, cohort 1	213	3.2 (2.8–3.7)	172	2.9 (2.5–3.4)	University of Bern
8. Selects 2015 Candidate survey	185	2.8 (2.4–3.2)	164	2.8 (2.4–3.3)	FORS
9.VoxIt: standardized post-vote surveys	124	1.9 (1.6–2.2)	106	1.8 (1.5–2.2)	Universities of Geneve and Zurich, FORS
10. Swiss Volunteering Survey 2016	120	1.8 (1.5–2.2)	109	1.9 (1.5–2.3)	University of Bern
Total	2651	39.8***	2158	36.9***	

* Traced from FORSbase online catalogue.

** Bootstrapped 95% stability intervals based on 1000 resamples

*** Column sums differing from cell sums are due to rounding

The most downloaded dataset, SHP Data Waves 1–19, is the Swiss annual household panel study based on a random sample of private households in Switzerland, interviewing all household members mainly by telephone. SHP is provided free of charge from FORSbase for the scientific community [57]. Other datasets are related with Swiss elections or popular votes (datasets 2, 3, 4, 5, 6, 9) or with education and civil society (datasets 7, 10).

The fact that the share of the ten most downloaded datasets decreases slightly if duplicates and versions of the same dataset are excluded (Table 5 “Percentage of total downloads” vs. “Percentage of unique user-dataset downloads”) shows that the most downloaded datasets are updated more often than the other datasets. However, the ranking of the most downloaded datasets does not change substantially showing that duplicates and versions spread quite evenly across those highly downloaded datasets. The bootstrapped 95%-stability intervals (see Table 5, column 3 in brackets) show that the ranking consists of four parts: A clear leader (dataset 1) and a clear second place (dataset 2) followed by a middle part (datasets 3 to 8) and studies 9 and 10 form the fourth group.

### Users of the archive

During the examined time window, 2281 unique users downloaded data from FORSbase. These users are called as “active users” in Table 6. In February 2020, there were 6628 registered users in FORSbase. Thus, only a third of the registered users downloaded a dataset during the time window (note that to upload data, one needs to register as a user). One half of the active users downloaded only one dataset during the given time period (Table 6, column on the righthand side). One fifth downloaded two datasets and 28 per cent downloaded three or more datasets. There was a group of heavy users downloading more than 5 datasets (5% of the registered users and 13% of the active users). At the end of the scale, one user downloaded 149 datasets during the time window. The group of 306 users downloading at least five datasets combined more than half (51.7%) of all the downloads during the time window. On average, considering all registered users, one user downloaded one dataset, while considering only active users, a user downloaded 2.9 datasets.

**Table 6:** Number of downloads per user.

Number of Downloaded Datasets	Number of Registered Users	Percentage of Registered Users	Percentage of Active Users
0	4347	65.6	n/a
1	1187	17.9	52.0
2	457	6.9	20.0
3	210	3.2	9.2
4	121	1.8	5.3
5+	306	4.6	13.4
Total	6628	100.0	100.0*

* Column sums exceeding 100% are due to rounding

Looking at unique user-dataset downloads (Table 7), 58 per cent of the active users downloaded only one unique dataset whereas 21 per cent downloaded two and 22 per cent three or more. The group of heavy users (5+ downloaded datasets) amounts to 4 per cent of all registered users and 11 per cent of the active users. The person who downloaded most datasets downloaded 140 unique datasets. If only unique user-dataset downloads are considered, the average is 0.9 downloads per registered user and 2.6 downloads per active user.

**Table 7:** Number unique dataset downloads per user.

Number of Downloaded Datasets	Number of Registered Users Downloading Unique Datasets	Percentage of Registered Users Downloading Unique Datasets	Percentage of Active Users Downloading Unique Datasets
0	4347	65.6	n/a
1	1311	19.8	57.5
2	474	7.2	20.8
3	160	2.4	7.0
4	95	1.4	4.2
5+	241	3.6	10.6
Total	6628	100	100*

* Column sums exceeding 100% are due to rounding

A clear majority of users downloaded only quantitative datasets (99%), 8 users downloaded both quantitative and qualitative data and 4 users only qualitative data.

Regarding the role of users, the majority of the downloads were made by users registered as students, while doctoral students, lecturers/postdocs and professors and other researchers were downloading less, and teachers and non-academics the least (Table 8).

**Table 8:** Frequency and share of registered user roles.

User group	Frequency	Percent
Student	3874	58.2
Doctoral student	954	14.3
Lecturer / post-doc	603	9.1
Professor	513	7.7
Other researcher, project manager	403	6.1
Teacher	196	2.9
Non-academic	113	1.7
Total	6656	100.0

**Fig 3:**
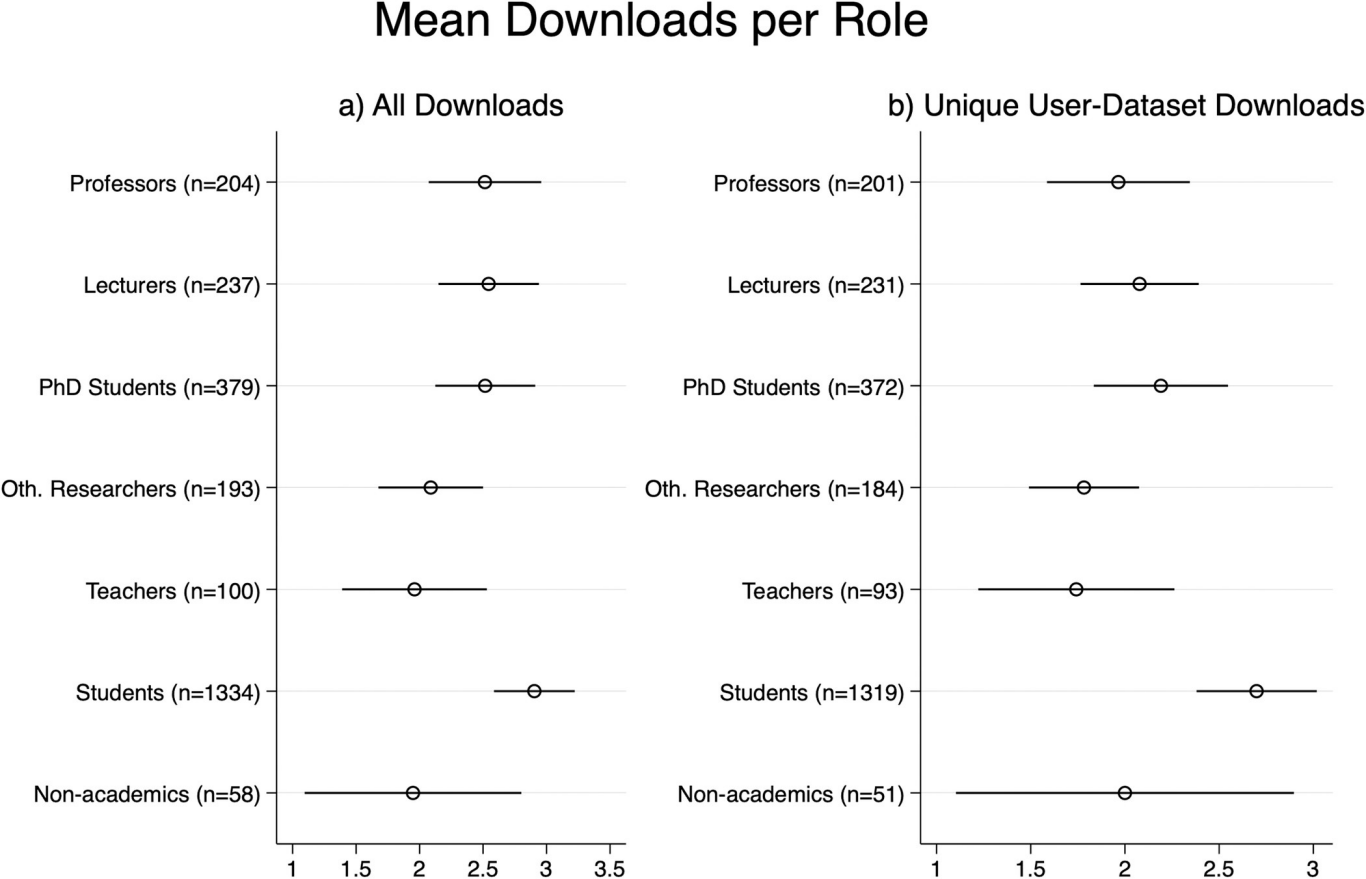
Average number of Downloads per user group with bootstrapped 95% stability intervals using 1000 resamples on the basis of a) all downloads and b) only unique user-dataset downloads.

Regarding download frequency across user groups, students were more likely to download many datasets compared to scholars, teachers, and non-academics (see Fig 3). Note that using bootstrapped regression, only the difference between students and scholars, teachers and non-academics were significant. If one takes only unique user-dataset downloads into account, students downloaded significantly more unique datasets than all other groups except for non-academics (as the latter have a large variability). However, the user roles are not clear-cut entities as the same person can indicate a different role for each download. This means that for unique user-dataset downloads only the first role is retained.

### Purpose of the downloads

The majority of the downloads were made for studying purposes (see Table 9). Of those downloading data for study purposes, at least 13 per cent (n = 497) downloaded the dataset for a bachelor’s thesis and at least 12 per cent (n = 452) for master’s thesis (combining 14.3% of all downloads used for a BA or MA thesis). However, these numbers represent minima because not all users did describe their purpose of download in such detail and the users not describing the purpose in detail might have used the data for a thesis as well.

**Table 9:** Purpose of the download.

Purpose of download	Frequency	Percent
Studying	3878	58.3
Research	2565	38.5
Teaching	213	3.2
Total	6656	100

Almost 40 per cent of the downloads served research purposes. Out of downloads used for research, at least 5 per cent download data for doctoral thesis (2% of the total downloads). However, the real share of downloading data for doctoral theses is probably much higher since more than 14 per cent of the users were registered as doctoral students.

Finally, only 3 per cent of the downloads served teaching purposes. This is surprising given that the biggest user group are students, and one would expect that it is the teachers who inform students about the dataset(s) used in the courses. However, users can only indicate one purpose for the download but can of course use it for many purposes after download. Also, it might mean that some teachers invite students to download the data themselves, while others download it and distribute the data to the students–which would mean that even more users would be students as the data covers only those students who downloaded the data themselves.

Users downloading datasets were also asked if they expect to write publications using the downloaded dataset. This was asked only if they were indicating that they were using the data for research and not teaching. Also, the question has a high share of non-response (463 or 7% of those who indicated research as the use of the download). Of those who replied to the question, a large majority (77.4%) did not expect to publish and just over one fifth expected to do so. Those downloading the dataset for research purposes were most likely to expect to write a publication (43%). Expectedly, professors, lecturers/postdoctoral researchers, and doctoral students expected publication more often compared to students (Table 10). Indeed, professors, lecturers/post-docs and, more unexpectedly, non-academics have a similar percentage intending to publish as the bootstrapped differences are not significant. All other groups do differ significantly from these three groups and between each other. The relationship between role and intention to publish is quite strong with a Cramér’s V of 0.43.

**Table 10:** Expecting publication of data download by user group.

User role	Percentage intending to publish	Bootstrapped 95% Stability Intervals
Professor (n = 513)	47.2	42.9–51.5
Lecturer / post-doc (n = 603)	48.1	44.0–52.3
Doctoral student (n = 954)	40.5	37.5–43.5
Other researcher project manager (n = 403)	31.8	27.4–36.4
Student (n = 3343)	7.2	6.4–8.1
Non-Academic (n = 96)	52.1	42.2–61.8
Total (N = 5912)	22.6	Cramér’s V = 0.43

Note. Bootstrapped stability intervals were calculated using 1000 resamples.

## Discussion

This study investigated whether there is a demand for open data in the social sciences by examining the use and users of a research data archive. It continued a discussion started by Late and Kekäläinen [15] studying the use of social science research data archives based on user log data. The results show that there is a demand for research data as datasets have been downloaded frequently from the FORSbase, i.e., on average 145 downloads per month. As in Finland [15], the number of downloads has increased in Switzerland from 2016 to 2019. During the time window of the study, a large majority (65%) of the datasets archived in FORSbase were downloaded at least for once. The share of downloaded datasets was similar with the Finnish results (70%) [15].

An overwhelming majority of the downloaded datasets are quantitative. The number of archived qualitative datasets in FORSbase is very low, which explains the low numbers in the downloads. Earlier studies have discussed the obstacles of data sharing and re-use in social sciences [38–40, 58]. Our results suggest that there might be strong differences in the habit of downloading open data from repositories across different specialisations: in qualitative social sciences, data sharing seems to be far less prominent than in quantitative social sciences. There is little evidence about the re-use of qualitative datasets and further studies are needed to understand the potential and pitfalls of open data policies for qualitative studies [53, 58]. The lack of data sharing, and re-use has certainly several reasons but ethical issues play an important role [59].

In this study, from the 725 archived datasets, the ten most frequently downloaded ones were investigated in more detail. Each of these datasets was downloaded more than 100 times, the most popular being downloaded more than 600 times. The downloads of these ten datasets amounts to almost 40 per cent of all downloads from the archive, which indicates that, similar to publications [60], a small share of datasets gains most of the attention. The same phenomenon was observed by Late and Kekäläinen [15]. The most frequently downloaded datasets share a few properties: all of them are longitudinal or time-series survey data collected not by individual scholars or research groups but by organizations or consortia such as FORS. Also, those datasets are local survey projects and the analysed archive, FORSbase, is the main source for obtaining this data. International longitudinal or time-series datasets were not among the ten most downloaded, even though local versions of these datasets would be available in the archive. Researchers interested in those cross-national datasets are more likely to download the datasets containing data from several countries from the international repository. Again, these results are in line with the study of Late and Kekäläinen [15]. In Finland, most downloaded datasets were local and national surveys. However, in the Finnish archive, the most downloaded datasets also included large international statistics collected by a single scholar. Qualitative datasets were also more often downloaded from the Finnish archive compared to the Swiss archive.

The fact that the most downloaded datasets were collected by prestigious and well-known organizations is in line with the argument raised in earlier studies [5, 9] that scholars’ trust in data is essential for the data re-use. However, what is considered as trustworthy may differ between disciplines. For the social scientists, reputation along with data selection and cleaning process play an important role in trust creation [61]. Systematic documentation and providing high quality paradata (i.e. data about the data) is valued by the data users [8, 9, 12, 62]. Other factors influencing the users’ trust in the data archives are recommendations, frequency of use, past experiences, and perceptions of the role of the archive [10]. However, frequently downloaded datasets are probably more well-known and thus, more visible for the users. Data findability is another critical point for data re-use that should be supported better [12, 52]. Furthermore, archives can increase their own visibility and prestige by archiving high quality and well-known datasets by establishing collection strategies and profiling for certain topics and data types to gain competitive advantage and reputation. However, the value of non-used (or non-downloaded) datasets cannot be overlooked, since they may become valuable in the future as needs are difficult to predict (i.e. delayed recognition in science [63]).

Earlier studies have not investigated the number of users of the data archives although it can be considered as an is important metric for evaluating the impact of archives. Our results show that FORSbase was used by more than 2000 unique users as one third of the registered users downloaded data from FORSbase. Most of them downloaded only one dataset. However, there was a smaller group of heavy users of the archive downloading several datasets and forming a remarkable share of all downloads. This might be an indication of field specific differences; in some fields of social sciences data can and is re-used more often. Also, it might indicate personal differences between the users. Users that have found datasets useful come back for downloading more relevant data or new versions of the datasets. Indeed, other studies have shown that scholars sharing their data are also more active re-users of data shared by others [12]. Our results show, however, that not all registered users download data which might indicate that some users of FORSbase use it for archiving, not data retrieval. Late and Kekäläinen [15] showed that users represented several countries, disciplines, and organisations. Our data did not allow for such analyses.

Earlier research has focused mainly on scholars’ data sharing and re-use practices and shown experienced scholars being the most active data re-users [12]. Yet, our findings confirm the results by Late and Kekäläinen’s [15] that students form the largest user group for the data archive. Students as a special user group should be taken into special consideration by data archives and service providers since there is a great potential in this user group as future data users and providers. Re-using data is important for developing knowledge creation skills and in socializing into the discipline [48]. Novice users have specific needs for data re-use and are influenced by experiences of their mentors [8]. Therefore, data archives need to pay special attention when thinking what services could be offered especially for the students and what guidance students need. More research, for example on the data management skills of students, is certainly needed. This is not only relevant for students who want to become future academics, but data becomes an important part of many professions in a digitalised society and skills in data use, management, archiving, and documenting will be relevant competences students need to learn. Also, scholars wish training for data management skills [64]. The role of data archives along with data managers and libraries have been identified as central in fostering such skills [17].

Only three per cent of the downloads served teaching purposes. However, studies by Late and Kekäläinen [15] and Bishop and Kuula-Luumi [53] show higher share of downloads for teaching purposes from Finnish and UK archives. There might be several reasons for the difference. However, users of FORSbase can only indicate one use purpose per download, while they could use the data for several purposes. Researchers can download a dataset for a research project and then use this project and the dataset in teaching without re-downloading the data and register it as a purpose for teaching. Also, they may ask the students to download the data, for example, in a research methods seminar. The high share of students among the users suggests that teaching is a frequent use of the datasets downloaded from FORSbase. However, an important question for future research is what data re-use means in teaching. Is it rather to teach research methods or also to replicate studies and foster the idea of responsible research already in teaching? Familiarizing students with the open research infrastructures might be an effective way to promote open science ideals.

More than one third of the downloads were made for research purposes. The share of research use was lower in the study by Late and Kekäläinen [15] covering only on fifth of the total use. In the Swiss archive, about half of the downloads for research were expected to result in a publication. Professors, lecturers, and post-doctoral scholars were most likely to plan to use the dataset for a publication. However, there is little evidence about how often re-used data are actually utilized in publications and for what purposes data are used for [65]. Unfortunately, no further information is available from our data that shows other research purposes than publications. Regarding Responsible Research and Innovation, it would be interesting to follow how often data is re-used for validation or replication purposes rather than publication.

Regarding the policy demand for open science and open data, the valorisation of data sharing becomes relevant. Data stewardship is not yet a relevant aspect in academic career development, which might hinder the motivation to share and document data sufficiently [36, 39]. However, European guidelines for responsible research assessment have already included data and data sharing as research outputs and activities to be recognized in the evaluation [66]. Therefore, further efforts should be made to study how (and how often) re-used datasets are cited in publications and how archives guide users to cite data. Data citation practices in social sciences are still evolving since citations are shown to be often incomplete or erroneous [15, 67–69]. Not all re-used research data are cited, at least not in a formal way [15]. Developing more formal data citation practices would enable a quantitative evaluation of the impact of data re-use. The challenge is to get scholars to cite data in a systematic way [70]. This would also serve the need to provide quantitative metrics for evaluating the impact of research infrastructures [6]. User log data can provide information concerning the number of downloaded data, but for evaluating the impact on research, further studies are needed exploiting, for example, bibliometric methods.

### Practical implications and limitations of the study

The results provide several practical implications for utilizing user log data for evaluating digital data archive use and as a source of research data. First, it would be important for the archives to define clearly what a data “version” is and to separate updates from new waves that comprise a new dataset. As new versions and updates of the datasets influence user behaviour and the number of downloads and thus, should be taken into consideration when user log data is used in archive evaluation or in research. The most frequently downloaded datasets are characterised by various versions and are updated more often than datasets provided by individual scholars. In our study we decided to analyse both, the full number of downloads and unique downloads to recognize the share of duplicates. The differences were not significant yet existed. Further, our results provide implications for collecting user log data. For example, information collection should cover all kinds of users and use types. In the case of FORSbase, for example, “studying” as a data re-use purpose was not provided. This underlines the importance of user studies for the service providers to truly know who their clients are. Given the relevance of replicational and open research data in science policy and the lack of knowledge on open research data practices, it is also advisable to archives to collect meaningful log data to be able to supplement ethical considerations with empirical evidence on data re-use.

This study comes with limitations: making conclusions about data re-use based on user log data is somewhat unreliable since it is likely that not all downloaded datasets are used, or some are used for many times or for other purposes than expected. Generalizing findings across organizations may be challenging because download metrics may be contingent on the specific characteristics of the data archive or related organisations [4]. For example, datasets can be used as course material possibly leading to hundreds of data downloads [15]. Additionally, log-data cannot provide qualitative insights into the data re-use (e.g., why a dataset was selected and how it was used). Still, user log data can give useful insights into re-use of research data and the users of data archives on the macro level beyond self-reported data re-use and from the point of view of the archive [5]. Our findings show that data is downloaded for various purposes and by various user groups from the archive. Thus, studying data re-use based for example on citations captures only part of the data re-use. Results of this study will give grounds for future studies in this respect. In addition, we analysed log data only from one archive. However, as our results are in line with a similar study conducted in Finland [15], we believe the results can be generalised to similar national social science data archives. Future research will show how the frequency of data downloads will develop as open data practices establish in the social sciences.

## Conclusions

This study contributes to our understanding of the utilization of digital data archives in the realm of social sciences. The findings indicate the demand for social science data, as evidenced by the increasing number of data downloads from a Swiss data archive. However, it is noteworthy that as majority of the archived datasets were downloaded at least once, a limited set of longitudinal and time-series survey datasets compiled by organizations rather than individual scholars gained substantial share of the downloads. Since the case archive primarily specializes in housing quantitative data, the re-use of qualitative data was marginal. Among the users, students constituted a significant proportion who accessed the archive to acquire data for their educational purposes. Nonetheless, the user base encompassed individuals from diverse roles, including experienced and novice scholars and non-academics. As the findings are in line with previous research [15] it is likely to find similar patterns across data archives specialised for the social sciences. The increasing availability of digital datasets for the re-use may create new data practices within social sciences.

Enriched log data capturing the use of the digital data archive provide a macro level understanding about the re-use of the data from singular archive. To obtain more comprehensive insights into data re-use and evolving data practices within social sciences, future research applying quantitative and qualitative approaches is needed. A future research agenda on data re-use would include comparative studies of different archives (which would preclude some previous agreement on the collection of meta-data between archives), studies into the (epistemological and empirical) meanings and definitions of re-use of research data in social sciences and into the trade-offs between collecting new data versus re-using existing data. A very important issue is the data citation practices in social sciences. For further developing the research infrastructures, user studies are needed to address how users interact with the infrastructures, what obstacles they face and what support they desire.

## Supporting information

S1 Data (XLSX)
